# Pediatric Scoliosis in Osteogenesis Imperfecta: From Genetic Mechanisms to Therapeutic Strategies

**DOI:** 10.1111/os.70170

**Published:** 2025-09-24

**Authors:** Vladislav Muldiiarov, Keely Buesing, Maegen J. Wallace

**Affiliations:** ^1^ Department of Surgery, Division of Acute Care Surgery University of Nebraska Medical Center Omaha Nebraska USA; ^2^ Phoenix Children's Hospital Phoenix Arizona USA

**Keywords:** genes, kyphosis, osteogenesis imperfecta, scoliosis, spine, vertebral deformity

## Abstract

Osteogenesis imperfecta (OI) is a hereditary connective tissue disorder characterized by increased bone fragility and a propensity for multiple fractures, often leading to various skeletal deformities. Spinal involvement, particularly the development of scoliosis, is one of the most serious clinical manifestations of OI, significantly impacting patients' quality of life. Scoliosis in OI is characterized by early onset and rapid progression, complicating its treatment and necessitating special attention. This review article consolidates the results of contemporary molecular‐genetic studies on spinal deformities in children with OI and examines the risk factors for their progression. It provides an overview of existing methods for treating scoliotic deformities in OI, including surgical and conservative approaches, and discusses prospects for the implementation of new therapeutic strategies. The aim of the review is to enhance the understanding of the pathogenesis of spinal deformities in OI and to contribute to the development of more effective methods for their diagnosis and treatment.

## Graphical abstract

This review explores the genetic underpinnings and treatment strategies for spinal deformities in children with osteogenesis imperfecta. Emphasis is placed on emerging molecular insights and modern surgical techniques that improve the management of severe scoliosis and enhance patient outcomes.

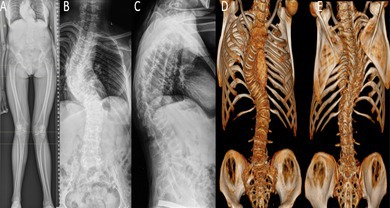

## 1 Introduction

Spinal involvement in osteogenesis imperfecta (OI) holds particular clinical significance. Progressive curvatures lead to loss of balance, chest deformities, pain syndrome, and impaired lung function, ultimately requiring surgical stabilization, usually during puberty [1, 2]. Severe spinal deformities including deformed and reduced vertebrae, extremely fragile bones, chest deformities, and short, deformed trunks as well as associated problems such as cervical spine anomalies and cranial base abnormalities (basilar impressions, cervical kyphosis), and deformed lower and upper limbs create numerous challenges in treatment [3, 4, 5, 6]. Early diagnosis and timely treatment of spinal deformities are critically important for preventing irreversible consequences and improving disease prognosis. Despite significant advances in understanding the pathogenesis of OI and in developing treatment methods, managing complex spinal deformities in this patient group remains a challenging task. Difficulties arise not only from the characteristics of the disease itself but also from the limitations of existing therapeutic approaches [7, 8].

This review presents modern concepts of diagnosis, treatment, and prevention of complex spinal deformities in young patients with severe OI. We will explore the results of recent molecular‐genetic studies, risk factors for the progression of deformities, and discuss prospects for the introduction of new therapeutic strategies, including innovative surgical and conservative methods.

## 2 Method

**FIGURE 1:**
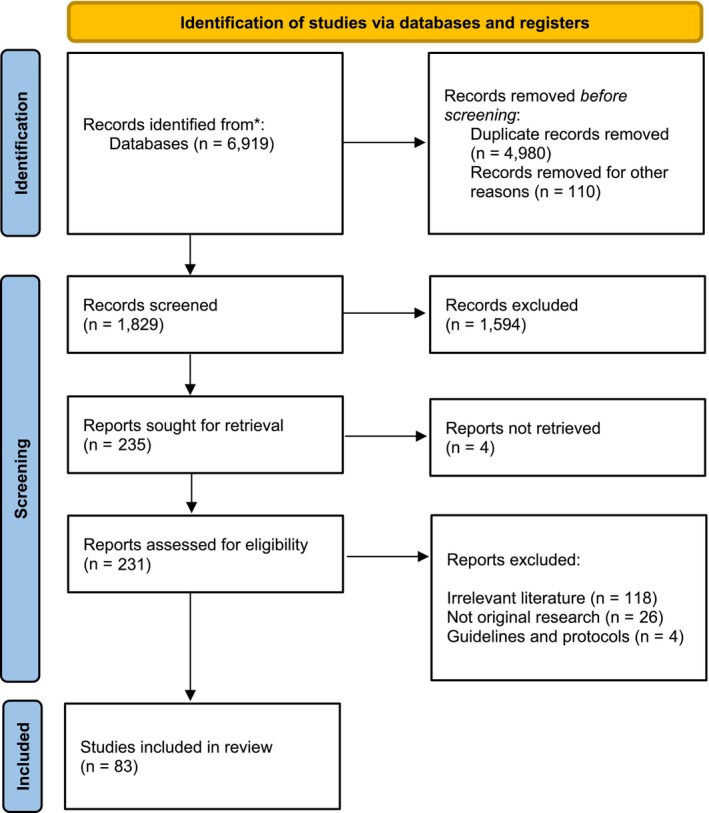
Study flow diagram.

This review was conducted in accordance with the PRISMA (Preferred Reporting Items for Systematic Reviews and Meta‐Analyses) guidelines, including a structured screening flowchart (Figure 1).

### 2.1 Search Methods for Identification of Studies

A comprehensive literature search was performed in PubMed, Web of Science, and Embase, supplemented by targeted searches in Google Scholar. The search strategy incorporated the following Boolean keyword combination: (“osteogenesis imperfecta” OR “OI” OR “brittle bone disease”) AND (“scoliosis” OR “spinal deformity” OR “genetics” OR “surgical treatment” OR “treatment” OR “risk factors” OR “gene”). Only English‐language publications available up to March 2025 were considered.

### 2.2 Literature Inclusion and Exclusion Criteria

Studies were selected based on predefined eligibility criteria.

Inclusion criteria were: (1) original research studies, systematic reviews, or meta‐analyses published in peer‐reviewed journals; (2) investigations addressing the genetic, clinical, or therapeutic aspects of spinal deformities in individuals with OI; and (3) studies employing in vitro, in vivo, or clinical models relevant to OI‐related scoliosis.

Exclusion criteria included: (1) studies not directly related to OI or spinal deformities; (2) editorials, guidelines, consensus statements, or conference abstracts; and (3) publications lacking extractable or analyzable data (e.g., sample size < 3, absence of outcome measures, or incomplete methodology), as well as reports exclusively describing lethal forms of OI were excluded.

After duplicate removal, two independent reviewers (VM and KB) screened titles and abstracts using the eligibility criteria. Articles excluded during this stage were categorized based on the reason for exclusion. Disagreements were resolved through discussion with a third reviewer (MW). Full‐text articles were then retrieved and evaluated using the same inclusion and exclusion criteria to determine final eligibility for analysis.

## 3 Genetic Influence on the Development of Spinal Deformities

OI has traditionally been classified into four types (I–IV) based on clinical, radiological, and hereditary data proposed by David Sillence [9]. Type I is a mild form characterized by blue sclerae, childhood fractures, and moderate bone deformities. Type II is a severe form, often lethal in the perinatal period, accompanied by multiple fractures and significant bone deformities. Type III is a severe form with multiple fractures, epiphyseal “popcorn” changes, short stature, deformities of long tubular bones, kyphoscoliosis, osteoporosis, blue sclerae, dentinogenesis imperfecta, and often hearing loss. Type IV is a moderate form with white or gray sclerae, frequently leading to short stature and possible hearing loss. This classification remains the cornerstone for the diagnosis and understanding of OI.

In 2004, Glorieux and Rauch expanded the classification by adding types V–VII, which include forms with unknown genetic defects and varying inheritance patterns: OI type V has presumed autosomal dominant inheritance, while types VI and VII are autosomal recessive [10]. Currently, the genetic classification encompasses 22 types of OI, allowing for a more detailed consideration of the disease's diverse manifestations [11, 12].

Given the variety of OI types and their clinical presentations, the prevalence of spinal deformities among patients with OI ranges from 25% to 100% [13, 14, 15]. In a study by Anissipour et al. involving 316 patients with OI, it was demonstrated that patients with more severe forms, especially type III, exhibit the highest rates of scoliosis progression—approximately 6° per year. Moreover, early bisphosphonate therapy initiated before the age of six can significantly slow curvature progression, reducing the curve progression rate by 3.8° per year. In contrast, patients without bisphosphonate therapy showed progression rates of 1° per year for type I OI and 4° per year for type IV OI. Bisphosphonate treatment started after 6 years of age or in patients with type I or IV OI did not have a statistically significant effect on scoliosis progression [16, 17]. A study by Sato et al. also demonstrated a significant impact of intravenous bisphosphonate therapy on slowing scoliosis progression, but only in patients with type III [16]. However, despite the deceleration of Cobb angle progression, this therapy did not prevent the development of moderate or severe scoliosis in adulthood. Thus, the treatment did not significantly reduce the prevalence of significant deformities.

This variation in scoliosis prevalence can be attributed to distinct genetic profiles and differences between Eastern and Western OI patient populations. In a study involving 560 patients, Lin et al. demonstrated that biallelic mutations or COL1A2 mutations were associated with more severe bone deformities and reduced mobility compared to COL1A1 mutations (all *p* < 0.05). Glycine substitutions in COL1A1 or COL1A2, as well as biallelic variants, resulted in more severe skeletal phenotypes. In contrast, haploinsufficiency of type I collagen α‐chains led to milder skeletal phenotypes [18].

Additionally, in a nationwide cohort of 1,596 OI patients with 1:1 propensity score matched controls (matched for age and follow‐up duration), Park et al. reported a 3.91‐fold higher prevalence of scoliosis in OI than in controls (20.8% vs 5.3%; *p* < 0.001) [19].

This underscores the necessity for a deeper understanding of genetic and other factors influencing the severity and progression of scoliosis in OI. A recent study by Chen et al., involving 290 patients, confirmed the importance of both genetic and non‐genetic factors in the severity and progression of scoliosis in OI [20]. It was found that genotypes and types of mutations significantly affect the clinical course of the disease. Specifically, mutations in the COL1A2 gene were less detrimental compared to those in COL1A1, whereas mutations in IFITM5 and WNT1 had a more pronounced negative impact. Additionally, nongenetic factors such as age and bone mineral density played crucial roles in the progression of deformities. The average rate of scoliosis progression in the studied cohort was 2.7° per year (95% confidence interval 2.4–3.0).

Beyond the aforementioned genes, studies of mutations in FKBP10 have opened new avenues in understanding the genetics of OI. Patients with homozygous variants in this gene exhibited moderately severe disease with progressive kyphoscoliosis, deformities of long bones, and gray sclerae. These mutations, which encode the FKBP65 protein, are associated with recessive type XI OI, Bruck syndrome, or Kuskokwim syndrome [21, 22, 23, 24, 25, 26].

Similarly, type XIII OI, caused by pathogenic variants in the SP7 gene, is characterized by underdevelopment of long bones and moderate scoliosis [27, 28]. In contrast, patients with type XVII OI are born with a normal skeleton but, in early childhood, display severe progressive OI with frequent fractures and muscle hypotonia [29, 30]. Type XX OI is a severe or lethal recessive skeletal dysplasia characterized by profound intellectual disability and progressive scoliosis, caused by mutations in the MESD gene [31, 32].

Furthermore, emerging data highlight the significance of mutations affecting the structure or functionality of molecules involved in TGF‐β signaling pathways. These defects can lead to scoliosis and other connective tissue disorders, such as Marfan syndrome, Ehlers‐Danlos syndrome, Loeys‐Dietz syndrome, and Shprintzen‐Goldberg syndrome. These syndromes are accompanied by severe musculoskeletal abnormalities, including pronounced scoliotic deformities [33, 34, 35]. A concise overview of OI types associated with clinically significant scoliosis and other spinal deformities is summarized in Table 1.

**TABLE 1:** Summary of osteogenesis imperfecta types and spinal deformity characteristics.

OI type	OMIM	Genetic characteristics	Clinical characteristics	Scoliosis prevalence (Number of patients studied) Spinal deformity characteristics
I	166200	COL1A1 AD	Mild, nondeforming. Most individuals have normal height, minimal deformities, blue sclerae, and hearing loss in 50% of cases. Dentinogenesis imperfecta, when present, is strongly hereditary	17.6% (244) Patel et al. [13] 36% (188) Sato et al. [16] 39% (159) Anissipour et al. [17] Mild scoliosis progressing 1–2 degrees per year, with codfish vertebrae in adults [13, 17, 36].
III	259420	COL1A1/COL1A2 AD	Bones are progressively deforming, often moderately at birth. Relative macrocephaly is present, with a scleral hue that usually lightens with age.	47% (100) Patel et al. [13] 89% (82) Sato et al. [16] 68% (81) Anissipour et al. [17] Progressive kyphoscoliosis, codfish vertebrae, and platyspondyly [13, 16, 17].
IV	166220	COL1A1/COL1A2 AD	Mild to moderate bone deformity with relative macrocephaly. Scleral hue is often bluish at birth, lightening with age. Higher risk of basilar invagination.	31.3% (147) Patel et al. [13] 61% (167) Sato et al. [16] 54% (59) Anissipour et al. [17] Progressive scoliosis up to 4° per year, with codfish vertebrae [13, 17, 36].
V	610967	IFITM5 AD	Variable scleral hue, forearm interosseous membrane calcification, radiodense metaphyseal bands at long bone growth plates, and radial head dislocation.	57% (30) Rauch et al. [37] 50% (28) Tan et al. [38] Moderate scoliosis with age‐related progression; vertebral fractures reported in up to 90% of patients [37, 38, 39, 40].
VI	613982	SERPINF1 AR	Moderate to severe skeletal deformity with variable scleral hue. Hearing loss and dentinogenesis imperfecta are absent.	55.6% (18) in patients > 10 years, Selina et al. [41] 27.3% (11), Patel et al. [13] Moderate scoliosis. Bone shows a “fish‐scale” pattern under polarized light and excessive osteoids in childhood [41, 42].
VII	610682	CRTAP AR	Severe or lethal bone dysplasia resembling types II and III, with small head circumference, exophthalmos, and white or light blue sclerae.	50% (8) Ward et al. [43] 40% (5) Patel et al. [13] Scoliosis (mild to moderate); vertebral fractures in up to 62.5% [13, 43].
XI	610968	FKBP10/FKBP65 AR	Progressive deforming dysplasia, grayish‐white sclerae, normal hearing, ligamentous laxity, joint hyperextensibility, and coxa vara.	63% (38) scoliosis developed in adolescence and often progressed rapidly. Schwarze et al. [44] 33% (19) had kyphoscoliotic deformity before treatment, 66% at final follow‐up [45]
XII	613849	SP7 AR	Mild bone deformities, generalized osteoporosis, delayed teeth eruption, progressive hearing loss, no dentinogenesis imperfecta, and white sclerae.	100% (12) across all reported cases, with scoliosis ranging from moderate to severe [28, 36]
XIII	614856	BMP1 AR	Severe form with normal teeth, pale blue sclerae, significant growth deficiency, and borderline osteoporosis	45% (20) across all reported cases of moderate scoliosis [46, 47, 48]
XV	615220	WNT1 AR	Moderate to severe; progressively deforming. Bluish to blue sclerae in some. Marked deformity, bowing of long bones.	80% (30) Li et al. [25]; 50% (20) Chen et al. [20]—scoliosis ranged from mild to moderate [49, 50]
XVII	616507	SPARC AR	Severe. White sclerae, no dentinogenesis, and joint hyperlaxity.	Up to 100% (5) prevalence of scoliosis and vertebral compression fractures [51].
XIX	301014	MBTPS2 XLR	Severe. Congenital limb bowing, early fractures increasing in childhood, blue sclerae, hypermobility, and motor delay.	100% (8) had kyphoscoliosis and multiple vertebral fractures [52].
XX	618644	MESD AR	Severe or lethal form. Dental anomalies, frequent intellectual disability, predominant rhizomelia/micromelia, and blue sclerae.	33% (9); all reported patients had multiple vertebral fractures [31, 32].
XXI	619131	KDELR2 AR	Severe. Bone fragility, short stature, joint hypermobility, normal/mildly discolored sclerae, reduced bone density, frequent fractures	56% (9); all reported patients had moderate to severe scoliosis [53, 54].
XXII	619795	CCDC134 AR	Severe bone fragility, significant growth deficiency, joint laxity, and early‐onset fractures.	33% (6) with severe scoliosis among all reported cases [55, 56].

Abbreviations: AD, autosomal dominant; AR, autosomal recessive; BMP1, bone morphogenetic protein 1; CCDC134, coiled‐coil domain containing 134; COL1A1, collagen, type I, alpha‐1; COL1A2, collagen, type I, alpha‐2; CRTAP, cartilage‐associated protein; FKBP10, FK506‐binding protein 10; IFITM5, interferon‐induced transmembrane protein 5; KDELR2, KDEL Endoplasmic Reticulum Protein Retention Receptor 2; MBTPS2, membrane bound transcription factor peptidase, site 2; MESD, mesoderm development LRP chaperone; OMIM, online Mendelian inheritance in man; SERPINF1, serpin peptidase inhibitor, clade F, member 1; SPARC, secreted protein, acidic, cysteine‐rich; XL, x‐linked.

Therefore, further investigation into genetic factors and their association with the clinical manifestations of scoliosis in OI will enable a deeper understanding of the disease's pathogenesis and facilitate the development of predictive diagnostic systems.

## 4 Risk Factors for the Progression of Spinal Deformities

### 4.1 Vertebral Fractures and Morphological Risk Factors

**FIGURE 2:**
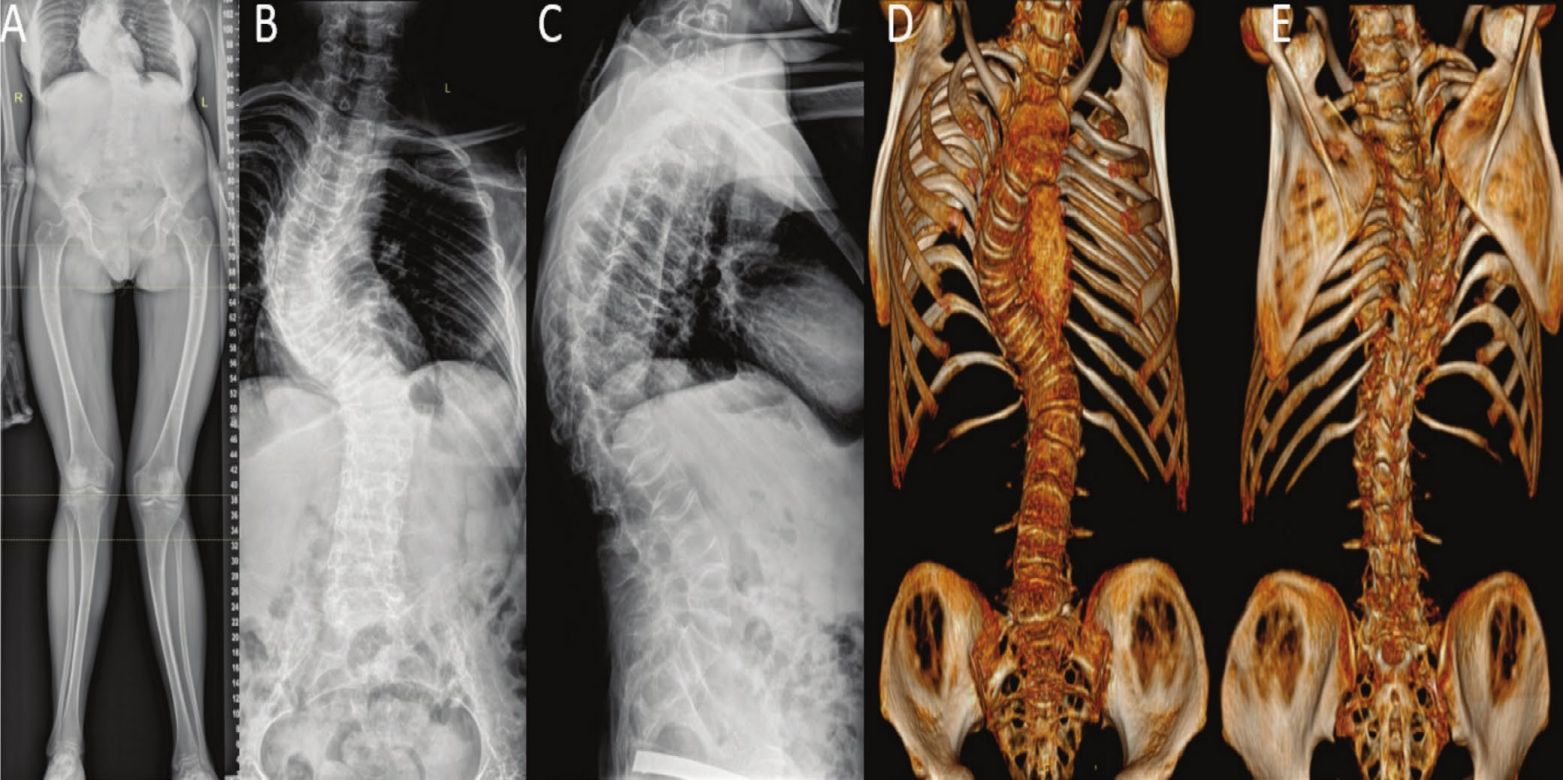
PA (A, B) and lateral (C) radiographs of a 17‐year‐old patient with type IV osteogenesis imperfecta demonstrating a 49° thoracolumbar scoliosis and a 51° thoracic kyphosis with osteopenic, wedge‐shaped, and biconcave vertebral bodies. (D, E) Additional 3D CT images are provided. Images published with permission from patient and parents. IRB approval not applicable.

Compression fractures of the vertebrae are common in patients with OI, particularly during childhood and adolescence when fracture risk is highest. Although the relative risk of fractures in patients with OI decreases with age compared to the general population, vertebral fractures can still occur in up to 71% of individuals with mild forms of the disease [57, 58]. The shape of the vertebrae can indeed be a significant risk factor for the development of spinal deformities [59] (Figure 2).

In a study by Ishikawa et al., 44 patients with OI were analyzed to determine the prevalence of spinal deformities [60]. The results showed that scoliosis was present in 68% of patients and kyphosis in 41%. The authors found that the presence of six or more biconcave vertebrae before puberty significantly increased the likelihood of developing severe scoliosis (> 50°). Supporting these findings, Abelin et al. observed increased thoracic kyphosis in children with OI. Interestingly, this enhanced kyphosis in the T1–T12 segment was noted even in the absence of significant changes in the T4–T12 segment, suggesting that thoracic kyphosis in patients with OI more often manifests in the upper thoracic spine [61]. Despite these risks, recent studies indicate that bisphosphonate therapy improves vertebral integrity and reduces variability in vertebral shape, which may decrease the risk of developing spinal deformities [62, 63, 64].

### 4.2 Biomechanical Stress and Motor Development

Another important factor contributing to the progression of deformities is increased mechanical loading during childhood. In the context of osteopenia, such loads can lead to bone remodeling and progressive deformities, with elongation of the vertebral pedicles being the most common outcome. Some cases of OI with severe hyperlordosis have been reported to be due to elongation of the lumbar vertebral pedicles and spondylolisthesis [65]. Beyond mechanical loading, neuromuscular development and connective tissue properties have also been explored as potential contributors to spinal deformity progression. The significance of early motor development is also emphasized in research. Engelbert et al. found that the age at which scoliosis first appears is associated with the age at which antigravity motor skills, such as “supported sitting,” are achieved [66].

In parallel, ligamentous laxity has been implicated in the pathogenesis of spinal deformities in other dysplastic conditions. For example, in Ehlers‐Danlos syndrome, hypermobility of the ligamentous apparatus is a well‐established predictor of scoliosis [67]. However, the evidence for a similar association in OI remains inconclusive. Several studies have failed to establish a consistent link between isolated musculoskeletal hypermobility and spinal deformities in pediatric patients with OI [68, 69].

### 4.3 Lung Function and Skeletal Fragility

Given the increased mortality associated with respiratory disorders in patients with OI, controlling and managing scoliosis becomes extremely important to prevent a decline in lung function. A cohort study in Denmark demonstrated that patients with OI have a threefold higher risk of death from respiratory diseases compared to the general population [70]. Previous studies have established that scoliosis characteristic of OI correlates with decreased lung function. It is suggested that severe scoliosis and spinal deformities in patients with OI can significantly impair pulmonary function [71, 72]. A study by Widmann et al. demonstrated that patients with Cobb angles greater than 60° had a significant reduction in vital capacity (on average down to 41% of the expected value). In contrast, patients with Cobb angles less than 60° had a vital capacity averaging 78% of the expected value [73]. Further supporting this, a study by Keuning et al. analyzed 42 patients with type III OI, all of whom had scoliosis with an average curve magnitude of 66°. The reduction in vital capacity correlated with the degree of scoliosis, especially with increased thoracic curvature. The authors concluded that the deterioration of lung function in these patients is associated both with the presence of scoliosis and with intrinsic characteristics of OI itself [74].

However, recent studies have shown that restrictive lung disease is not exclusive to patients with severe spinal deformities [75, 76]. Some individuals with OI who have mild scoliosis or none at all exhibit similar pulmonary outcomes to patients with more pronounced scoliosis [77, 78].

Moreover, a study by Yonko et al. (*n* = 157) on the quality of life in OI showed that respiratory symptoms negatively affect psychosocial well‐being and limit daily physical activity. These limitations arise regardless of the type of OI, age, or presence of scoliosis, reflecting the significant impact of respiratory status on the quality of life of patients with OI [79]. A review conducted by Storoni et al. concluded that pulmonary insufficiency in patients with OI has a combined etiology. They suggested that abnormal type I collagen distorts the intrapulmonary structure, while skeletal dysplasia causes severe chest wall abnormalities, exacerbating respiratory disorders [80].

Recent studies have confirmed this, showing that lung tissue abnormalities in OI have an intrinsic etiology that can be exacerbated by scoliosis. All patients with type III OI and half of those with type IV exhibited restrictive lung function, and many had parenchymal defects such as atelectasis and reticulation [81]. As a result of functional defects directly related to collagen abnormalities, 90% of patients in the studied group showed reduced gas exchange.

## 5 Management of Spinal Deformities in Children

The primary goals of treatment in OI include reducing fracture frequency, alleviating pain, and supporting the growth, mobility, and functional independence of patients. As previously noted, the degree of limitations and treatment approaches depends on the severity of the disease. Effective management of OI requires a multidisciplinary team involving physiotherapists, medical geneticists, orthopedic surgeons, and other specialists depending on specific symptoms and potential complications.

### 5.1 Pharmacologic and Conservative Approaches

Bisphosphonates are potent inhibitors of bone resorption that reduce osteoclast activity, thereby decreasing bone turnover. Numerous studies have demonstrated that the use of bisphosphonates can improve vertebral body height loss when started at a young age [82, 83, 84, 85]. However, retrospective studies have shown that bisphosphonates have a limited effect on reducing the incidence of scoliosis in children with OI as they grow older. Although bisphosphonates do not reduce the frequency of scoliosis, they can slow disease progression in patients with type III OI. Moreover, the reduction in vertebral compression allows for the use of pedicle screws for more reliable fixation during surgical correction of scoliosis [16]. Regarding treatment with monoclonal antibody drugs in children, research is limited due to a common and dangerous side effect—rebound hypercalcemia. However, an important prospective study conducted by Lui et al. involved 84 children with OI who were randomized to receive subcutaneous injections of denosumab every 6 months or a single intravenous infusion of zoledronic acid. Vertebral height and projection area significantly increased after both denosumab and zoledronic acid treatment [86].

Additionally, sclerostin inhibitors such as romosozumab and setrusumab, representing a novel class of monoclonal antibodies, are currently being investigated in an international clinical trial to evaluate their effectiveness in children with OI [87, 88].

The effectiveness of conservative treatment of existing structural deformities using exercises or custom‐made braces remains unclear. No changes in the natural course of the disease have been observed, which is not surprising. Considering the fragility of the ribs that transmit corrective forces to the spine, it is logical to assume that the use of a brace may be problematic or even harmful. Preexisting chest deformities may complicate the proper fitting of the brace, cause excessive corrective forces, exacerbate the already altered chest geometry, and even negatively affect respiratory function and soft tissues. In several studies conducted, braces failed to halt disease progression in most patients to whom they were prescribed [1, 2, 6, 89].

### 5.2 Surgical Treatment of Scoliotic Deformity

After the diagnosis of scoliosis, patients are recommended to undergo regular monitoring every 6–12 months to track curve progression through examinations and radiography when clinically necessary. Similar to idiopathic scoliosis, identified risk factors for curve progression in patients with OI include residual growth and the magnitude of the curve. Most surgeons recommend surgical treatment for scoliosis greater than 50° to stop curve progression and prevent pulmonary complications [7].

One of the first major mentions of surgical treatment of scoliotic deformity in patients with OI was by Cristofaro et al., who studied 49 patients with OI and found that 71% of them had scoliosis with an average curve magnitude of 42° [6]. Bracing failed to prevent curve progression, leading to surgical intervention in eight patients. Of these, five underwent instrumented fusion with Harrington rods, and three received non‐instrumented posterior fusion. While all patients achieved radiographic fusion within 10 months postoperatively, no meaningful improvement in spinal curvature or ambulatory function was observed.

Subsequently, Yong‐Hing and MacEwen presented a multicenter study of 121 patients with OI, 60 of whom underwent some form of spinal fusion; most received Harrington rod instrumentation, and some were fused in situ without instrumentation [1]. They achieved a 36% correction of the Cobb angle in the group of 60 patients, with a 7% better correction in those treated with Harrington rods compared to the group without instrumentation. However, this early instrumentation led to complications in more than 50% of cases, which were also associated with preoperative curve magnitude and the degree of kyphosis. Benson recommended improving the Harrington construct by supplementing hooks with methylmethacrylate bone cement [90].

Janus et al. reported the treatment outcomes of 20 patients with severe scoliosis in OI who underwent preoperative halo traction and posterior spinal fusion with instrumentation [91]. Preoperative traction improved the Cobb angle of scoliosis by 32% and kyphosis by 24%, with slight loss of correction during follow‐up. Complications were rare, and seven out of 20 patients showed improvements in walking and functional abilities.

Sienko et al. conducted the largest study on scoliosis in OI, covering 2372 patients. They found that scoliosis was present in 429 patients, and only 17.2% of them required surgical intervention, with 8% needing reoperation on average 3.88 years later. The prevalence of scoliosis among OI types corresponded to the trends mentioned above [92].

Cement augmentation has proven effective in increasing the strength of pedicle screws in adult patients with osteoporosis. Applying bone cement around the screws enhances pullout strength and extends the lifespan of the construct under cyclic loads [93, 94, 95]. Biomechanical studies show that using polymethylmethacrylate for augmentation can increase screw pullout strength by 110%–190%. Additionally, coating screws with hydroxyapatite enhances pullout resistance and reduces the risk of fixation loosening [96, 97, 98]. Yilmaz et al. described their experience using cement‐augmented pedicle screw fixation in seven patients with OI. In this retrospective series, there were no complications directly related to cement fixation, and significant improvements in apical vertebral rotation were noted [99]. Despite the advantages, the use of bone cement can be accompanied by complications such as cement leakage into the spinal canal and the risk of embolism [100, 101, 102].

Modern segmental pedicle screw fixation provides rigid stabilization, three‐column spinal control, and force distribution along the construct, and has become the advanced method in managing OI‐related spinal deformities [103].

In summary, surgical correction of scoliotic deformities remains a cornerstone of care for patients with OI. Recent advances in spinal instrumentation, especially the adoption of segmental pedicle screw fixation combined with cement augmentation, have expanded treatment possibilities by providing stronger fixation and more consistent deformity correction. Preoperative halo traction can further optimize outcomes by improving curve flexibility and reducing intraoperative challenges. Although these approaches have shown promising results, complications such as cement leakage and the potential need for reoperation underscore the ongoing need for refined techniques and vigilant patient monitoring.

## 6 Limitations and Future Directions

This review has several limitations that reflect broader challenges in the existing literature. The populations studied are often heterogeneous, with variability in OI subtypes, age at diagnosis, prior bisphosphonate exposure, and surgical indications. These factors complicate direct comparisons between studies and limit the ability to draw generalized conclusions. In addition, much of the available evidence is derived from retrospective, single‐center case series with small cohorts and short follow‐up durations, which reduces statistical power and external validity. The lack of standardized outcome measures further impairs the ability to synthesize findings across studies, particularly with respect to functional status, pulmonary outcomes, and quality of life. Investigations into novel therapies, such as monoclonal antibodies and sclerostin inhibitors, remain limited in scale and duration, precluding definitive assessments of long‐term efficacy and safety.

To address these limitations, future research should emphasize well‐designed prospective studies and coordinated multicenter trials that incorporate standardized data collection protocols. The development of international registries with robust genetic, radiographic, surgical, and functional data would facilitate the identification of prognostic markers for deformity progression and surgical risk. These initiatives are critical for advancing personalized treatment strategies, improving surgical planning, and optimizing long‐term outcomes in children with OI.

## 7 Conclusion

It is recommended to consider the genetic background of patients with OI to assess strategies for managing spinal deformities in children. Early use of bisphosphonates helps strengthen bone tissue and reduce the risk of fractures. The main indications for surgical treatment are a deformity angle greater than 50° according to the Cobb method and progression of the deformity by more than 4° per year. The primary challenges in surgical correction of spinal deformities in these patients are associated with poor bone quality and rigidity of the deformities. Modern fixation systems using pedicle screws provide reliable stabilization and allow for three‐dimensional correction control. Undoubtedly, a multidisciplinary approach combining pharmacological treatment, surgical correction, and rehabilitation therapy significantly improves functional outcomes, enhancing the quality of life and functional abilities of patients with OI.

## Author Contributions

**Vladislav Muldiiarov:** writing – review and editing, writing – original draft, software, project administration, visualization, conceptualization. **Keely Buesing:** writing – review and editing, resources, project administration, visualization, methodology. **Maegen J. Wallace:** conceptualization, writing – review and editing, supervision, resources, project administration, methodology.

## Conflicts of Interest

The authors declare no conflicts of interest.
